# The proximal first exon architecture of the murine ghrelin gene is highly similar to its human orthologue

**DOI:** 10.1186/1756-0500-2-85

**Published:** 2009-05-09

**Authors:** Inge Seim, Shea L Carter, Adrian C Herington, Lisa K Chopin

**Affiliations:** 1 Institute of Health and Biomedical Innovation, Queensland University of Technology, Kelvin Grove, Queensland, Australia

## Abstract

**Background:**

The murine ghrelin gene (*Ghrl*), originally sequenced from stomach tissue, contains five exons and a single transcription start site in a short, 19 bp first exon (exon 0). We recently isolated several novel first exons of the human ghrelin gene and found evidence of a complex transcriptional repertoire. In this report, we examined the 5' exons of the murine ghrelin orthologue in a range of tissues using 5' RACE.

**Findings:**

5' RACE revealed two transcription start sites (TSSs) in exon 0 and four TSSs in intron 0, which correspond to 5' extensions of exon 1. Using quantitative, real-time RT-PCR (qRT-PCR), we demonstrated that extended exon 1 containing *Ghrl *transcripts are largely confined to the spleen, adrenal gland, stomach, and skin.

**Conclusion:**

We demonstrate that multiple transcription start sites are present in exon 0 and an extended exon 1 of the murine ghrelin gene, similar to the proximal first exon organisation of its human orthologue. The identification of several transcription start sites in intron 0 of mouse ghrelin (resulting in an extension of exon 1) raises the possibility that developmental-, cell- and tissue-specific *Ghrl *mRNA species are created by employing alternative promoters and further studies of the murine ghrelin gene are warranted.

## Background

Ghrelin is a 28 amino acid peptide, predominantly expressed in the stomach, and is cleaved from a 117 amino acid preprohormone, preproghrelin [1]. Our previously published comparative genomics analysis suggested that the mouse and human first exon architecture is conserved [2] and we demonstrated that the human ghrelin gene (*GHRL*) contains several untranslated first exons that may play a role in regulating ghrelin gene translation [2]. In this study we investigate the existence of novel exons of the murine ghrelin orthologue (*Ghrl*).

## Findings

### Identification of a novel first exon and transcription start sites of the murine ghrelin gene

It has previously been reported that the mouse ghrelin gene consists of four coding exons (exons 1 to 4) and a short, non-coding 19 bp first exon [3], which we have termed exon 0. To determine if additional first exon and transcription start sites are present, 5' RACE (rapid amplification of 5' complementary DNA ends) was performed with exon 1-specific reverse primers and a RACE-ready panel of anchored cDNA libraries derived from 24 mouse tissues (OriGene, Rockville, MD). A list of exons and exon-intron boundaries of the ghrelin locus derived-transcripts identified in this and previous studies is given in [Additional file 1].

**Figure 1:**
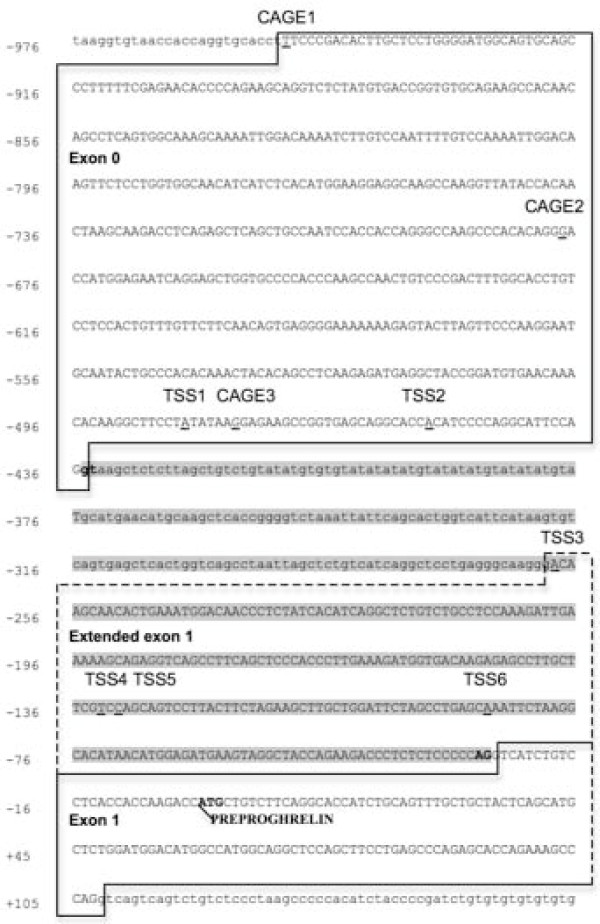
**Partial genomic sequence of the murine ghrelin gene showing exon 0 and extended exon 1 transcription start sites in a number of tissues**. Exon sequences are boxed and in upper case letters. The extended exon 1 sequence is indicated by dashed boxes. Nucleotide positions are shown on the left and the translation initiation site of preproghrelin is indicated as +1. Intron 0 is highlighted in grey and its intron boundaries are shown in bold letters. Six transcription start sites (TSSs), denoted TSS1-6 (underlined and indicated), were demonstrated using an OriGene Sure-RACE Multi-Tissue RACE panel. TSS1 initiates a 48 bp exon 0 (exon 0b) [GenBank:FJ355944]; TSS2 [GenBank:FJ355945] initiates a 19 bp exon 0 (exon 0a); TSS3-6 (exon 1e-b, respectively) all initiate transcripts with an extended exon 1 [GenBank: FJ355940–FJ355943]. The exon 0 CAGE tag starting sites T06R06CF33DC, T06R06CF32CB and T06R06CF3202 are termed CAGE1-3, respectively.

Sequencing of clones from the murine stomach, lung, and 9.5-day embryo revealed two transcription start sites (TSS) in exon 0 (Fig. 1), which are 48 bp [GenBank: FJ355944] and 19 bp [GenBank:FJ355945] in size. The 19 bp exon 0 (exon 0a) is identical to a sequence previously reported in the mouse stomach [3], while the 48 bp exon 0 (exon 0b) is flanked by a CAGE tag starting site (CTSS) (Fig. 1). CTSSs are obtained by large-scale sequencing of concatemers derived from the 5' ends of capped mRNA and indicate the transcription start site [4].

**Figure 2:**
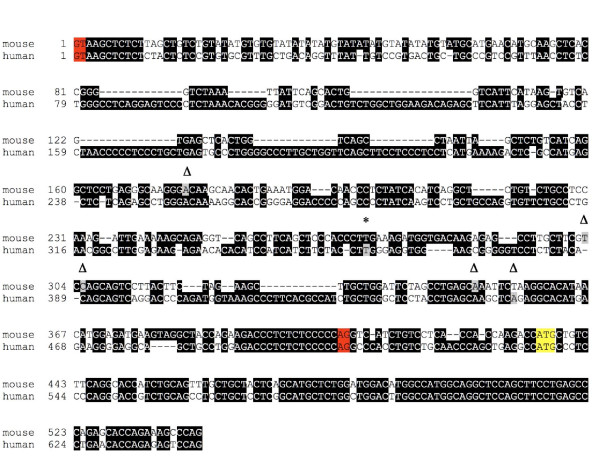
**Comparison of mouse and human ghrelin exon 1 sequences showing exon 1 extended transcription start sites**. The mouse and human alignments were generated by the ClustalW program and drawn by BOXSHADE ). Black shading indicates conserved nucleotides. Transcription start sites determined by 5' RACE (Δ), as well as a full-length cDNA clone [GenBank:BM982194] (*), are indicated, and the exact transcription start site nucleotides are shaded in grey in each species. Exon-intron boundaries are highlighted in red. The start codon of preproghrelin is highlighted in yellow.

In our earlier report we noted that a region immediately upstream of exon 1 (intron 0) of the ghrelin gene is highly conserved between the mouse and human orthologues [2]. This suggested that transcription of the murine ghrelin gene could also be initiated from within intron 0. We isolated murine 5' RACE clones that contained extended exon 1 sequence (Fig. 1). These clones were isolated from 9.5-day embryo, 12.5-day embryo, adult lung, and adult adrenal gland [GenBank:FJ355940–FJ355943] and correspond to four transcription start sites within intron 0 of the mouse ghrelin gene. Interestingly, an extended exon 1 that contains 61 bp of intron 0 sequence (exon 1e) is present in a conserved region immediately upstream of exon 1 [2] and is flanked by the previously reported human transcription start site [5] (Fig. 2).

In a recent study we also identified a human ghrelin exon (exon -1) 2.6 kb upstream of the preproghrelin translation start site in exon 1 [2]. These human exon 1-derived ghrelin transcripts contain a putative secretion signal peptide, which is not present in the rodent sequence, and may give rise to novel peptides [2]. Mouse ghrelin appears to lack exon -1, as we have been unable to identify murine exon -1 ghrelin sequence using 5' RACE and RT-PCR (data not shown). Promoter sequences are known to evolve rapidly and show a high rate of sequence turnover [6,7]; thus, it is not surprising that the region corresponding to murine exon -1 is not a functional ghrelin gene exon in the mouse.

### The extended exon 1 of *Ghrl *is transcribed and gives rise to full-length preproghrelin transcripts

**Figure 3:**
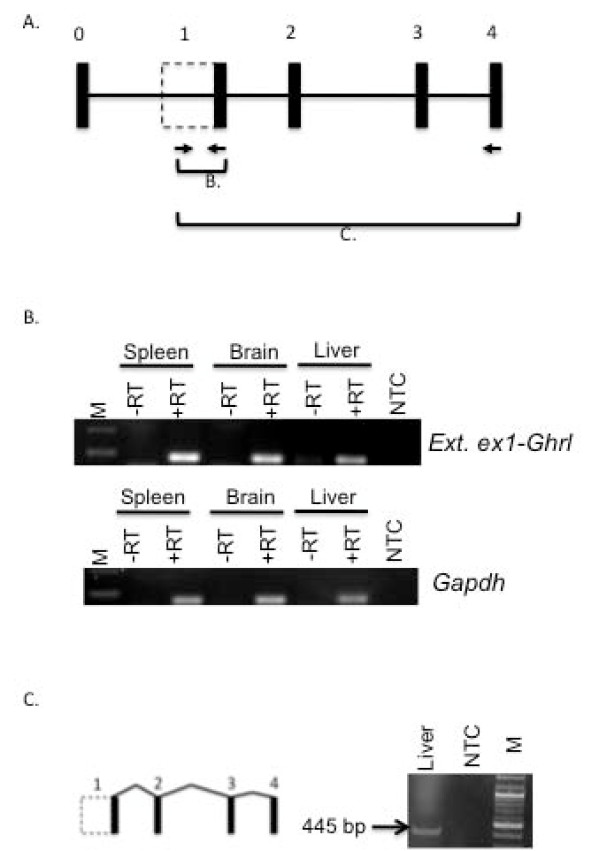
**Verification of an extended exon 1 in the mouse ghrelin gene**. **A**. Schematic diagram showing the structure of the murine ghrelin gene. Exons are represented as boxes and introns as horizontal lines. PCR primers employed in B. and C. are shown as arrows and the exon regions amplified are indicated. **B**. Ethidium bromide stained agarose gel electrophoresis demonstrating *Ghrl *extended exon 1 to exon 1 RT-PCR amplicons. DNase treated total RNA was amplified by RT-PCR with reverse transcriptase (+RT), or without (-RT) reverse transcriptase to demonstrate that there was no genomic DNA contamination. *Gapdh *was used as an endogenous control. **C**. Ethidium bromide stained agarose gel electrophoresis of a 445 bp full-length preproghrelin amplicon derived using primers located in the extended exon 1 (Ext1-F) and exon 4 (Ex4-R). M = 100 bp DNA molecular weight marker ladder (New England Biolabs). NTC = no template negative control.

While 5' RACE data [5] and sequence from a full-length cDNA clone (derived from primary cultures of cystic fibrosis lung epithelial cells) [GenBank:BM982194] demonstrate the existence of human full-length preproghrelin transcripts with an extended exon 1, there has been no evidence that this region is transcribed in the mouse. To verify the existence of the extended exon 1 we performed RT-PCR experiments (Fig. 3). RT-PCRs, employing a sense primer in the extended exon 1 and an antisense primer in exon 1, confirmed that exon 1 is transcribed and not a result of genomic or exogenous contamination (Fig. 3B). Next, we obtained an amplicon containing the extended exon 1 to 4 in the liver and spleen [GenBank:FJ914266], proving experimentally that the extended exon 1 can give rise to full-length preproghrelin transcripts (Fig. 3C).

**Figure 4:**
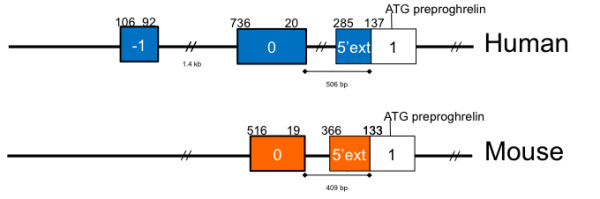
**Comparison of the human and mouse ghrelin gene**. The human ghrelin gene (*GHRL*) contains three first 5' untranslated exons (-1, 0 and a 5' extended exon 1/5'ext) shown as blue boxes. Note that the human exon -1 can also splice into exon 0. The murine ghrelin gene (*Ghrl*) contains exons homologous to the human exon 0 and the 5' extended exon 1 (shown as orange boxes), but lacks exon -1. The minimum and maximum size (in base pairs) of exons is shown above each exon.

It was previously reported that the murine ghrelin gene consists of five exons and a single transcription start site in a short, 19 bp exon 0 [3]. Our results demonstrate that the murine gene consists of at least two first exons (exon 0 and exon 1) (Fig. 4) with multiple transcription start sites within each first exon region, which is typical of broad-type promoters [8].

### Ghrelin transcripts with an extended exon 1 are highly expressed in a limited number of tissues

**Figure 5:**
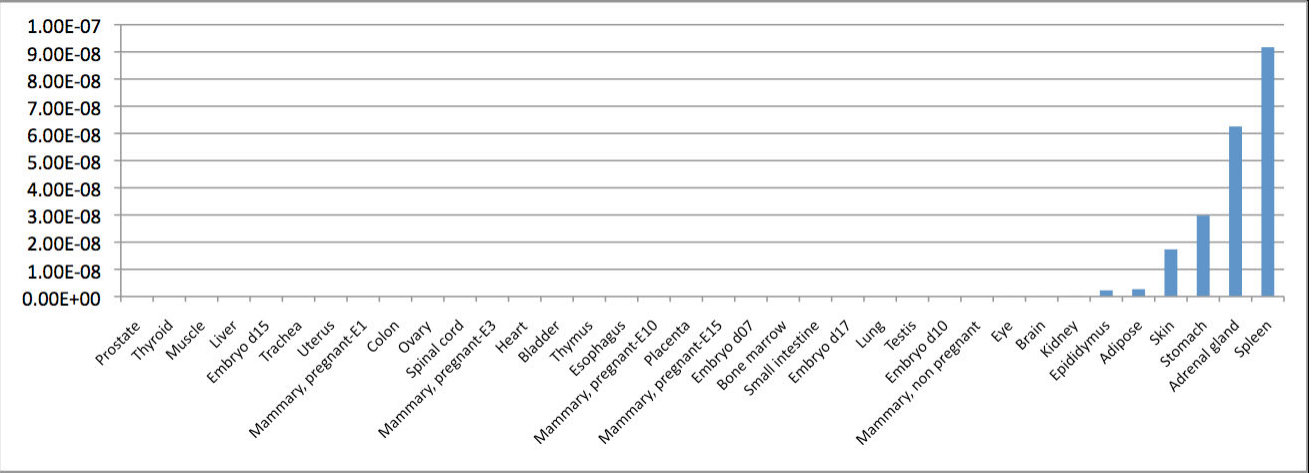
**Real-time RT-PCR demonstrating tissue-specific expression of murine ghrelin transcripts with an extended exon 1**. Relative expression of extended exon 1 of *Ghrl *in a wide range of mouse tissues (TissueScan Mouse Normal Tissue qPCR array). Calculations of *Ghrl *expression levels were performed using the standard curve method (correlating the threshold cycle number (C_T _values) and copy numbers of *Ghrl*) and normalised to the expression of *Gapdh*.

We also investigated the distribution of extended exon 1-containing *Ghrl *transcripts by transcript profiling in 36 normal mouse tissues. This analysis indicated that the extended exon 1 species are predominantly expressed in the spleen, adrenal gland, stomach, skin, adipose tissue, and epididymis (Fig. 5). Interestingly, the human extended exon 1 is also differentially expressed, with equal levels of the 20 bp exon 0 and extended exon 1 in the stomach [5]. In contrast, very high levels of the extended exon 1 and almost undetectable levels of the 20 bp exon 0 have been reported in the human thyroid medullary carcinoma TT cell line [5]. In both humans and rodents, total ghrelin mRNA levels are very high in the stomach, while much lower levels can be found in other tissues, including the adrenal gland [9,10], suggesting that the expression of the extended exon 1 may be developmental-, cell- and tissue-specific.

### A possible role for 5' variant exons of preproghrelin transcripts in translational control

As observed previously for exon 0 of the human ghrelin gene [2], several very short upstream open reading frames (uORFs) are present in the extended exon 1 and exon 0 sequence (data not shown). Upstream open reading frames, mRNA secondary structure and other motifs in 5' untranslated exons have been shown to regulate the translation of developmental genes [11]. Interestingly, it has been reported that ghrelin mRNA and protein levels do not directly correlate in the rat [12]. We hypothesise that this may be caused by the transcription of different first *Ghrl *exons with different preproghrelin translation efficiencies. This mechanism is exemplified in the chicken embryo where transcripts harbouring uORFs allow low-level translation of proinsulin, whereas a higher level of proinsulin expression is achieved in the adult pancreas by transcription of mRNAs from a downstream first exon devoid of uORFs [13]. Moreover, alternative 5' untranslated exons can have an mRNA secondary structure that restrains translation, particularly if a hairpin occurs close to the 5' cap, which is the ribosomal entry site [14]. The short 19 bp exon 0, which we and others [3] have described, is devoid of upstream open reading frames or stable secondary structure. This transcript, therefore, could be more efficiently translated than the 233 bp 5' extended exon 1 (exon 1e) that contains a 266 bp 5' untranslated region, for example (see [Additional file 2]).

## Conclusion

In this report, we demonstrate that transcription start sites in exon 0 an extended exon 1 are present in the murine ghrelin gene, suggesting that there are similarities in the proximal first exon organisation of the murine ghrelin gene and its human orthologue. A novel, extended exon 1 is expressed at high levels in the spleen, adrenal gland, stomach, and skin, indicating that the murine ghrelin gene harbours a cell-type, development-stage and/or tissue-specific, proximal promoter in intron 0. Little is known about the promoters of the murine ghrelin gene. Only the role of rat ghrelin sequence, which is upstream of the 19 bp exon 0, has been investigated in promoter constructs in murine cell-lines, and this demonstrated minimal promoter activity [15]. The upstream, proximal promoter region of the murine ghrelin gene, therefore, warrants further studies.

## Methods

### Bioinformatics

The murine ghrelin gene (*Ghrl*) architecture was examined using the RIKEN Genomic Element Viewer provided by the FANTOM (Functional Annotation of Mouse) consortium [16]. This database includes transcripts within the mouse genome as well as CAGE (Cap Analysis of Gene Expression) tags corresponding to the 5' ends of transcripts [16]. The location of ghrelin locus-derived ESTs and mRNA entries, as well as sequenced PCR amplicons obtained in this study, were identified by BLAST searches against GenBank databases [17]. The minimum free energies (ΔG) of the 5' untranslated regions (5' UTRs) were calculated using the RNAfold web server [18].

### 5' RACE

5' RACE was performed with primers specific for exon 1 of *Ghrl *using a mouse Sure-RACE kit (OriGene) according to the manufacturer's instructions. Samples of cDNAs from 24 murine tissues (brain, heart, kidney, spleen, thymus, liver, stomach, small intestine, muscle, lung, testis, skin, adrenal gland, salivary gland, uterus, prostate, embryonic developmental stages (8–9-day, 9.5-day, 12-day and 19-day post-conception), breast (immature, pregnant, lactating, involuting) were challenged. All tissues were from NIH Swiss mice, except for the breast tissues that were obtained from outbred CD-1 mice. An outer PCR was performed for 25 cycles at 62°C with a 5' RACE adapter-specific forward primer and a *Ghrl *exon 1-specific reverse primer (RACEout-F/R, Table 1). PCR products were then diluted 1/100 and subjected to a nested PCR with adapter and *Ghrl *exon 1 gene specific primers (RACEin-F/R, Table 1) at 61°C for 35 cycles. All PCRs were performed in a total reaction volume of 50 μl using 1 U of Platinum Taq Polymerase High Fidelity (Invitrogen) according to the manufacturer's instructions. PCR products were purified by ethanol purification, sub-cloned into *pGEM-T Easy *(Promega) and sequenced by the Australian Genome Research Facility (AGRF, Brisbane, Australia) using the ABI PRISM BigDye Terminator Cycle Sequencing Kit v3.1 protocol (Applied Biosystems, AB, Foster City, CA).

**Table 1:** Designations and sequences of primers used in RT-PCR

Name	Sequence (5'-3')	Ghrl Exon	Ta (°C)	PCR Cycles
RACEout-F	AATTCGTCACTCCGTGAATCAG	N/A		

RACEout-R	CAGAGCATGCTGAGTAGCAG	1	62	25

RACEin-F	GTCACTCCGTGAATCAGATCG	N/A		

RACEin-R	CAGCAAACTGCAGATGGTG	1	61	35

Ext1-F	AAGGCACATAACATGGAGATGAAG	1*		

Ex1-R	CTTGGTGGTGAGGACAGATGAC	1	60	40

Ex4-R	GCCTGTCCGTGGTTACTTGT	4	58	40

Gapdh-F	ACCTGCCAAGTATGATGACATCA	N/A		

Gapdh-R	GGTCCTCAGTGTAGCCCAAGAT	N/A	60	40

Annealing temperatures (Ta) of oligonucleotide primers employed in RT-PCR are shown. The location of oligonucleotide primers spanning ghrelin exons are listed, while oligonucleotides spanning synthetic sequences (adapters and linkers) or genes other than *Ghrl *are denoted as N/A (not applicable). The novel extended exon 1 is denoted as 1*. All primers were synthesised by Proligo (Armidale, Australia).

### Verification of extended exon 1 and cloning of full-length preproghrelin with an extended exon 1

2 μg DNase-treated total RNA from the liver, brain and spleen of Swiss Webster mice (Ambion) was reverse transcribed with 200 units of SuperScript III (Invitrogen) using oligo(dT)_20 _primers in a final volume of 20 μl according to the manufacturer's instructions. The resulting single-stranded cDNA was treated with ribonuclease H (Invitrogen). PCRs were performed with a forward primer in the extended exon 1 (Ext1-F, Table 1) and a reverse primer in exon 1 (Ex1-R, Table 1) in a total reaction volume of 50 μl using 1 unit Platinum *Taq *Polymerase (Invitrogen) according to the manufacturer's instructions. RNA samples that were not reverse transcribed were run to control for genomic DNA and a no template control (water) was included to control for exogenous contamination. Cloning of full-length (exon 1 to 4) *Ghrl *transcripts containing the extended exon 1 was achieved by PCRs using the same extended exon 1 primer (Ext1-F, Table 1) and a primer in exon 4 (ex4-R, Table 1). PCR products were purified and sequenced as described above.

### Transcript Profiling in Mouse Tissues

Transcript profiling of murine *Ghrl *transcripts that contain an extended exon 1 was performed by using a TissueScan Mouse Normal Tissue qPCR array (OriGene) derived from both male and female NIH Swiss mice. The samples in this array are generated by reverse transcription of mouse poly(A)^+ ^RNAs, which are free of genomic DNA, using oligo(dT) primers. The array plates are loaded with equal amounts of cDNA per well, as described by the manufacturer (OriGene). Extended exon 1 transcript expression levels were examined using 2× SYBR Green PCR Master Mix (AB) and the primers Ext1-F/R (Table 1). The amount of mRNA was determined by normalising the levels of expression with the glyceraldehyde 3-phosphate dehydrogenase (*Gapdh*) (Gapdh-F/R, Table 1) expression in the tissues. Primers were designed using Primer Express v.2.0 (AB) to yield a single amplicon, and were verified by dissociation curve analysis. The quantitative real-time RT-PCR conditions were optimised using purified PCR products. Real-time RT-PCR was performed using the 2× SYBR green PCR master mix on the AB 7000 sequence detection system and data analysed using the absolute standard curve method (User Bulletin #2, AB) to determine expression levels.

## Competing interests

The authors declare that they have no competing interests.

## Authors' contributions

IS conceived and designed the study and carried out all experiments except quantitative real-time RT-PCR. SLC carried out real-time RT-PCR. ACH and LKC participated in its design and co-ordination. All authors reviewed and approved the final manuscript.

## Supplementary Material

Additional file 1 **Compilation of exons and exon-intron boundaries of the murine ghrelin gene**. This is a PDF listing exons and intron boundaries of mouse ghrelin locus derived transcripts. Exon and intron sizes (bp) are indicated. Experimental evidence and/or references to the literature for each exon are shown. Note that exons 1b-e correspond to 5' extended exon 1 sequences. Click here for file

Additional file 2 **Compilation of 5' UTR sequences of the murine ghrelin gene**. Compilation of 5' UTR sequences of the murine ghrelin gene (*Ghrl*) based on 5' RACE clones obtained in this study. The length (AA) of putative upstream open reading frames (uORF) corresponds to the distance from the start codon of preproghrelin to the transcription start site. The minimum free energies (ΔG) of the 5' UTRs were calculated using the RNAfold web server [18]. The more negative the minimum free energy an RNA structure has, the more secondary structure the RNA is likely to have. Click here for file
