# Supplementary material for: The proximal first exon architecture of the murine ghrelin gene is highly similar to its human orthologue
Source: BMC Res Notes. 2009 May 9;2:85. doi: 10.1186/1756-0500-2-85 (PMC2689246; doi:10.1186/1756-0500-2-85)
Supplement: Additional file 1 — Compilation of exons and exon-intron boundaries of the murine ghrelin gene. This is a PDF listing exons and intron boundaries of mouse ghrelin locus derived transcripts. Exon and intron sizes (bp) are indicated. Experimental evidence and/or references to the literature for each exon are shown. Note that exons 1b-e correspond to 5' extended exon 1 sequences. [file 1756-0500-2-85-S1.pdf]

Additional File 1: Compilation of exons and exon-intron boundaries of the mouse ghrelin gene

Exon	Size (bp)	Comment	Intron size (bp)	Experimental evidence or external reference
0a	19	First exon	409 bp to exon 1	5’ RACE [1], RPA [1], RT-PCR [2]
0b	48	First exon		5’ RACE
0c	42	First exon		CAGE (T06R06CF33DC)
0d	243	First exon		CAGE (T06R06CF32CB)
0e	516	First exon		CAGE (T06R06CF3202)
1a	133		93 bp to exon 2	[1]
1b	366	First exon (233 bp 5’ extension of exon 1)		5’ RACE
1c	240	First exon (107 bp 5’ extension of exon 1)		
1d	238	First exon (105 bp 5’ extension of exon 1)		
1e	194	First exon (61 bp 5’ extension of exon 1)		
2	117		1942 bp to exon 3	[1]
3	109		783 bp to exon 4	
4	143	Terminal exon		

References

1. Tanaka M, Hayashida Y, Iguchi T, Nakao N, Nakai N, Nakashima K: **Organization of the mouse ghrelin gene and promoter: occurrence of a short noncoding first exon**. *Endocrinology* 2001, **142**(8):3697-3700.
2. Wei W, Wang G, Qi X, Englander EW, Greeley GH, Jr.: **Characterization and regulation of the rat and human ghrelin promoters**. *Endocrinology* 2005, **146**(3):1611-1625.
