# Supplementary material for: The proximal first exon architecture of the murine ghrelin gene is highly similar to its human orthologue
Source: BMC Res Notes. 2009 May 9;2:85. doi: 10.1186/1756-0500-2-85 (PMC2689246; doi:10.1186/1756-0500-2-85)
Supplement: Additional file 2 — Compilation of 5' UTR sequences of the murine ghrelin gene. Compilation of 5' UTR sequences of the murine ghrelin gene (Ghrl) based on 5' RACE clones obtained in this study. The length (AA) of putative upstream open reading frames (uORF) corresponds to the distance from the start codon of preproghrelin to the transcription start site. The minimum free energies (ΔG) of the 5' UTRs were calculated using the RNAfold web server [18]. The more negative the minimum free energy an RNA structure has, the more secondary structure the RNA is likely to have. [file 1756-0500-2-85-S2.pdf]

**Exon**	**5’ UTR length**	**uORFs**	**ΔG**	**Comment**
0b	76	0	-14.6	Splicing into exon 1 of *Ghrl* demonstrated by RT-PCR
0a	47	0	-4.27	
1b	261	3 (4, 15, 22 AA)	-69.9	
1c	135	1 (4AA)	-33.3	
1d	133	1 (4 AA)	-29.0	
1e	89	1 (4 AA)	-15.7	
